# A web-based educational therapy intervention associated with physical exercise to promote health in fibromyalgia in Brazil: the *Amigos De Fibro (Fibro Friends)* study protocol

**DOI:** 10.1186/s13063-023-07588-3

**Published:** 2023-10-09

**Authors:** Mateus Dias Antunes, Felipe Cayres Nogueira da Rocha Loures, Ingred Merllin Batista de Souza, Ariela Torres Cruz, Priscila de Oliveira Januário, Mara Maria Lisboa Santana Pinheiro, Ana Carolina Basso Schmitt, Elisa Frutos-Bernal, Ana María Martín-Nogueras, Amélia Pasqual Marques

**Affiliations:** 1 Program in Rehabilitation Sciences, Department of Physiotherapy, Speech-Language Pathology and Audiology, and Occupational Therapy, Faculty of Medicine, University of São Paulo, São Paulo, Brazil; 2 Clínica de Reumatismo Rocha Loures, Maringá, Paraná, Brazil; 3 Department of Statistics, Faculty of Medicine, University of Salamanca, Salamanca, Spain; 4 Faculty of Nursing and Physiotherapy, University of Salamanca, Salamanca, Spain

**Keywords:** Fibromyalgia, Chronic pain, Web-based intervention, Health promotion, Self-care, Patient education, Health education, Multicomponent intervention, Exercise, Primary health care

## Abstract

**Background:**

Health education is one of the main items to enable health promotion to patients with fibromyalgia. The objective of the study “Amigos de Fibro (Fibro Friends)” is to evaluate the impact of an educational intervention associated with physical exercise based on the web in promoting health and quality of life of patients with fibromyalgia in Brazil.

**Methods:**

A study with a randomized controlled trial approach will be carried out. The sample will consist of 24 participants, divided into two groups, with 12 individuals each. The experimental group will participate in meetings with lectures, debates, conversation rounds and exercises by a multidisciplinary team. Physical exercises will also be performed in an online environment. On the other hand, the control group will receive an e-book of education and self-care. Primary outcomes will be quality of life. The secondary outcomes will be sociodemographic and health profile, pain intensity, sleep quality, self-care agency, usage and costs of health and social care services, viability of the program and program participation. In addition, a qualitative evaluation process will be carried out with the participants. After the intervention, the data of both groups will be collected again, as well as after 3, 6, and 12 months to verify the effect and the maintenance of the intervention.

**Discussion:**

The results will provide data for studies to consider the use of this tool in the future by professionals working in the field of rheumatology.

**Trial registration:**

The protocol was registered in the Brazilian Registry of Clinical Trials RBR-3rh759 (https://trialsearch.who.int/Trial2.aspx?TrialID=RBR-3rh759). Date of registration: 07/02/2020].

## Administrative information

Note: the numbers in curly brackets in this protocol refer to SPIRIT checklist item numbers. The order of the items has been modified to group similar items (see http://www.equator-network.org/reporting-guidelines/spirit-2013-statement-defining-standard-protocol-items-for-clinical-trials/)

**Table:** 

Title {1}	A web-based educational therapy intervention associated with physical exercise to promote health in fibromyalgia in Brazil: the *AMIGOS DE FIBRO (FIBRO FRIENDS)* study protocol
Trial registration {2a and 2b}.	The protocol was registered in the Brazilian Registry of Clinical Trials RBR-3rh759 (https://trialsearch.who.int/Trial2.aspx?TrialID=RBR-3rh759).
Protocol version {3}	Version 1, Date: June 3, 2023
Funding {4}	This study was financed in part by the Coordenação de Aperfeiçoamento de Pessoal de Nível Superior – Brasil (CAPES) – Finance Code 001. This funding collaborated with a doctoral scholarship for the researcher in charge, contributing to the study design process and the collection, analysis and interpretation of data and writing of the manuscript. The promoted grant helps the researcher stay at the university, covering expenses with housing, food and academic research.
Author details {5a}	Mateus Dias Antunes^1^, Felipe Cayres Nogueira da Rocha Loures^2^, Ingred Merllin Batista de Souza^1^, Ariela Torres Cruz^1^, Priscila de Oliveira Januário^1^, Ana Carolina Basso Schmitt^1^, Elisa Frutos-Bernal^3^, Ana María Martín-Nogueras^4^, Amélia Pasqual Marques^1^ 1. Program in Rehabilitation Sciences, Department of Physiotherapy, Speech-Language Pathology and Audiology, and Occupational Therapy, Faculty of Medicine, University of São Paulo, Brazil. 2. Rheumatologist at the “Clínica de Reumatismo Rocha Loures” in the city of Maringá, Paraná, Brazil. 3. Department of Statistics, Faculty of Medicine, University of Salamanca, Salamanca, Spain. 4. Faculty of Nursing and Physiotherapy, University of Salamanca, Salamanca, Spain.
Name and contact information for the trial sponsor {5b}	Mateus Dias Antunes Program in Rehabilitation Sciences, Department of Physiotherapy, Speech-Language Pathology and Audiology, and Occupational Therapy, Faculty of Medicine, University of São Paulo, Brazil. Rua Cipotânea, 51, Cidade Universitária, CEP: 05360-000, São Paulo, SP, Brazil. Email: mateusantunes@usp.br Tel: +55 11 3091-7459
Role of sponsor {5c}	The funder is only responsible for the doctoral scholarship to the researcher in charge.

## Introduction

### Background and rationale {6a}

Fibromyalgia is one of the most common causes of generalized chronic musculoskeletal pain in the adult population. There is no consensus on the etiology or pathophysiology of fibromyalgia; many theories have been developed. However, it is generally accepted that fibromyalgia is a multifactorial health condition. That is, the symptoms of the syndrome develop and are maintained based on the interaction of several factors at the biological, psychological, and social levels [1]. The worldwide prevalence of fibromyalgia in the population is 0.2 to 6.6%. In Brazil, this prevalence is 2.5% [2], being higher in women, representing between 2.4 and 6.8%. In addition, in urban areas, prevalence reaches 0.7 and 11.4%, and in rural areas between 0.1 and 5.2% [3]. Studies also point to the presence of fibromyalgia in men [4–6]. Although the prevalence of fibromyalgia does not have the same notoriety as other rheumatologic conditions, its annual economic impact is considered very high [7, 8]. People diagnosed with fibromyalgia also make greater use of health services, especially in medical consultations in primary care and specialized care in several countries [1, 9].

The European Alliance of Associations for Rheumatology (EULAR) recommends the use of non-pharmacological therapies as a first-line intervention for patients with fibromyalgia, with patient education and physical exercise being an indispensable combination in the management of symptoms [10]. Physical exercise is one of the most accepted modalities. Studies have shown that physical exercise was well tolerated by patients with fibromyalgia. These studies achieved positive effects on pain intensity, fibromyalgia impact, hyperalgesia, physical function, catastrophizing, and psychological distress, without major adverse events [11–15]. Health education is one of the main items to enable the health promotion of patients with fibromyalgia. This strategy should prepare people to take control and responsibility for their own health and the health of their environment, as well as prepare them for empowerment, decision-making, participation, social control and action on the conditions, and determinants of their health and quality of life. Due to the chronic nature of fibromyalgia, patients deal negatively with this syndrome and its consequences and, to address this problem, educational programs that promote the self-management of the syndrome have been recommended to promote the health of people with fibromyalgia [16–18].

Self-management programs through educational practices are designed to help patients master the tasks needed to live with a chronic condition. Based on concepts of self-efficacy, they aim to increase a person’s confidence and ability to exercise control over worrisome health symptoms. Self-management programs have been identified as an effective means to improve quality of life and health functions, reducing the use of health resources [19–21]. Pain self-management interventions are recommended as an essential component of evidence-based clinical practice guidelines for persistent pain [21, 22].

Web-based group self-management interventions demonstrate better outcomes in small and specific populations of patients suffering from pain [20, 21, 23]. In addition, few studies to date have explored the role that web-based self-management programs can play in reducing fibromyalgia symptoms and increasing alternative methods of pain control [24]. High-quality studies are necessary to determine the effects of web-based interventions focusing on the therapeutic education of patients with fibromyalgia to promote health in primary health care in Brazil. Arnold and Clauw [25] highlighted the need to develop new biopsychosocial approaches for the treatment of fibromyalgia that focus on the management of symptoms in addition to pain through practices involving patient education. In addition, these authors highlight the lack of studies that measure significant clinical changes, which would allow a better clinical interpretation of the results of the investigation [1].

### Objectives {7}

The primary objective of the “Amigos de Fibro (Fibro Friends)” study is:

To evaluate the effectiveness of a web-based educational intervention associated with physical exercise to promote the health and quality of life of people with fibromyalgia in Brazil.

The secondary objectives are four:

1. To verify the effect of an educational intervention associated with physical exercise via the web on pain intensity, sleep quality, and self-care management in patients with fibromyalgia.
2. Measure the use and costs of social and health care services and the feasibility of a web-based educational intervention associated with physical exercise aimed at patients with fibromyalgia.
3. To identify the adherence of individuals with fibromyalgia participating in an educational program associated with physical exercise to promote health via the web.
4. Evaluate the maintenance of results obtained in individuals participating in an educational program associated with physical exercise for health promotion via the web in the short, medium, and long term.

### Trial design {8}

**Fig. 1:**
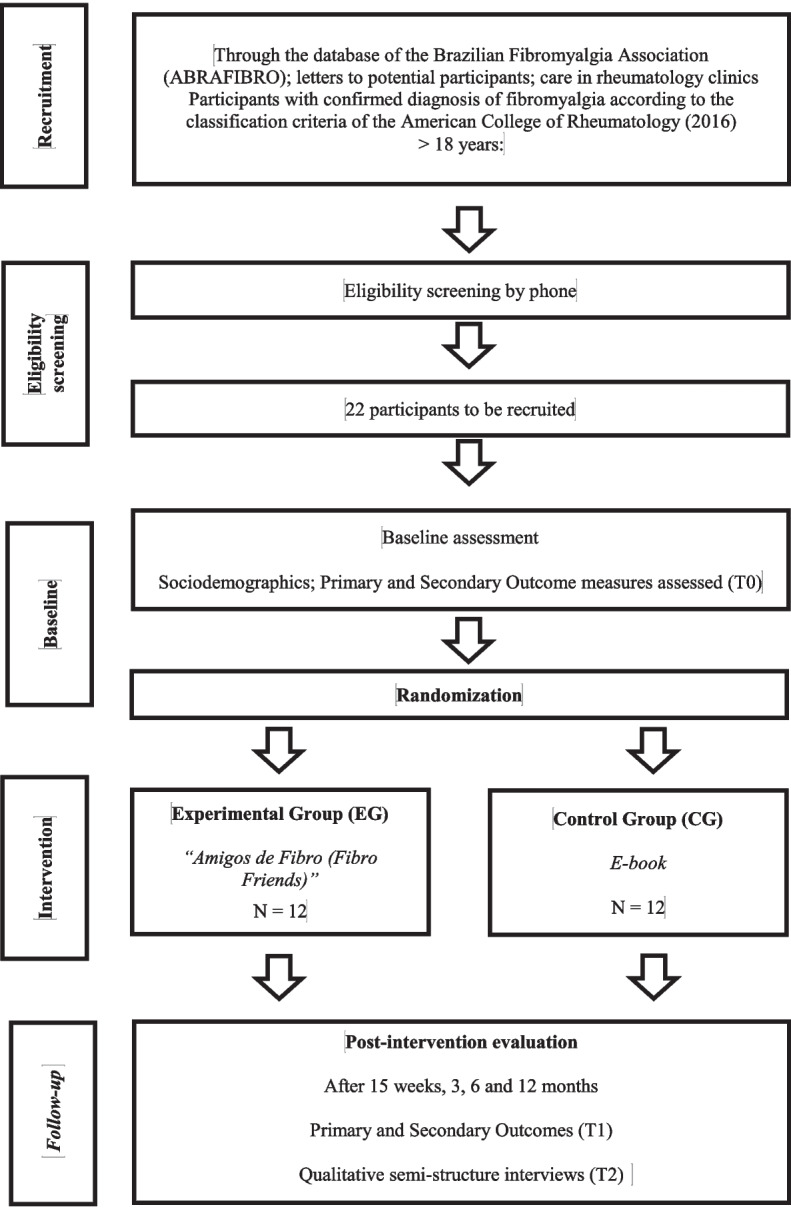
Flowchart of the study recruitment procedure

A study with a randomized controlled trial approach will be carried out. For the design of this study approach, a recommendation guide was considered, based on methodological points that must be followed in the textual description of the studies, the Consolidated Standards of Reporting Trials Statement (CONSORT). Objectivity and subjectivity cannot be thought of as opposites, as a quantitative study can raise questions to be deepened qualitatively, and vice versa. In this sense, a qualitative study will be carried out. Qualitative studies aim to relate the question of intentionality and meaning as inseparable from human actions, structures, and social relations. For the design of the qualitative study, the Consolidated Criteria for Reporting Qualitative Research (COREQ) was considered which is a checklist with evaluation criteria for qualitative research reports using interviews. This study is the first in Brazil and seeks to be a reference in the country so that in the future, it will be a model to be applied in all Basic Public Health Units in Brazil. Initial studies with these characteristics are recommended for studies focused on public health, in order to implement evidence-based interventions [26–30]. The flow of participants is shown in Fig. 1.

## Methods: participants, interventions and outcomes

### Study setting {9}

The study will be carried out through the Google Meet® online platform (a free and easily accessible videoconferencing platform developed by Google that allows users to host and participate in virtual meetings, video calls and webinars, offering features such as screen sharing and the ability to schedule and record meetings). The participant will be able to access it through smartphone, tablet, or computer in a synchronous way.

The study will be carried out in Brazil, allowing the participation of people from all states. In addition, the study is being carried out by researchers from the “Program in Rehabilitation Sciences, Department of Physiotherapy, Speech-Language Pathology and Audiology, and Occupational Therapy, Faculty of Medicine, University of São Paulo, Brazil”.

If necessary, participants may receive training or instructions for using the Google Meet® platform. This will be questioned before starting the study, on which the tool domain by the participant. Participants will be instructed to verify that the devices they will be using have access to the Google Meet® online platform and an internet connection.

Before starting the intervention, there will be a time agreed upon by the participants for the weekly meeting. In addition, participants will receive a weekly link to access the videoconference.

Furthermore, all participants will receive instructions to follow a Code of Conduct based on the Scientific Electronic Library Online (SCIELO). This code, guided by the principles and practices of Diversity, Equity, Inclusion and Accessibility, aims to create an open, healthy, and safe environment for all participants of an online event. All people involved in “Amigos de Fibro (Fibro Friends)” will be expected to be respectful and inclusive during their participation, avoiding threats of violence, the use of harmful, harmful or prejudiced language (including prejudice related to disabilities, race, nationality, sexual orientation, gender, age, among others), personal attacks, or any form of harassment. This type of behavior will not be tolerated, and participants who do not follow the code of conduct will be subject to being removed from the ongoing project and prevented from participating in future projects organized by those responsible for the research. If any participant has questions, witnesses violations of the code of conduct, or feels attacked, that is, a victim of harassment or any type of harassment, they will be instructed to contact the responsible researchers.

Finally, each meeting will be structured in at least three basic moments:

1. Initial moment of preparation of the group for the day’s work (engagement dynamics, warm-up, relaxation);
2. Intermediate moment in which the group got involved in varied activities, which facilitated their reflection and elaboration of the theme developed;
3. Moment of systematization and evaluation of the day’s work, which allowed participants to visualize their production as a work group.

### Eligibility criteria {10}

#### Inclusion criteria

The inclusion criteria will be as follows: literate individuals (those who can read and write) of both sexes, aged 18 years or older, with a medical diagnosis of fibromyalgia, proven by the International Statistical Classification of Diseases and Related Health Problems (ICD-10), with code M79.7, and confirmed by an evaluator according to the ACR Classification Criteria, revised 2016 version. In addition individuals with suitable electronic devices and internet access, preserved speech and hearing capacity, which allow the application of questionnaires.

#### Exclusion criteria

The exclusion criteria will be as follows: diagnosis of other conditions causing chronic pain (neuropathies, rheumatoid arthritis, osteoarthritis, spinal stenosis, or neoplasia); medically proven severe mental disorders (schizophrenia, psychosis, bipolar affective disorder, severe depression); individuals with visual or hearing impairments.

### Who will take informed consent? {26a}

Subjects will be instructed verbally and in writing about their participation in the study before signing the Informed Consent Form.

### Additional consent provisions for collection and use of participant data and biological specimens {26b}

This trial does not involve collecting biological specimens for storage.

## Interventions

### Explanation for the choice of comparators {6b}

Web-based educational intervention associated with physical exercise compared with simple educational material. The reasons for choosing interventions are according to the European Alliance of Associations for Rheumatology (EULAR), because it recommends the use of non-pharmacological therapies as a first-line intervention for patients with fibromyalgia, with patient education and physical exercise being a combination indispensable in the management of symptoms [10]. First, the experimental group comes from the purpose that physical exercise is one of the most accepted modalities. Studies have shown that physical exercise was well tolerated by patients with fibromyalgia. These studies achieved positive effects on pain intensity, fibromyalgia impact, hyperalgesia, physical function, catastrophizing, and psychological distress, without major adverse events [11–15]. In addition, health education is one of the main items to enable the promotion of the health of patients with fibromyalgia, because this strategy must prepare people to take control and responsibility for their own health and the health of their environment, as well as prepare them for empowerment, decision-making, participation, social control, and action on the conditions and determinants of their health and quality of life [16–18]. Regarding the choice of comparing with only one educational material (e-book), the proposal was due to the fact that the material has already been validated and published and showed a good level of agreement between the evaluators [31]. Thus, this type of intervention only includes health education and is not associated with physical exercise. Both programs (Amigos de Fibro and the e-book) were developed in Brazil by the authors. In addition, both are in Portuguese (Brazilian) language and are available in open access for consultation.

### Intervention description {11a}

#### Study groups

##### Intervention

The meetings will be organized by two physiotherapists, who will invite a multidisciplinary team working in primary care to give lectures and hold debates, rounds of conversation and exercises through the Google Meet platform. In addition, a physiotherapist will apply the physical exercises described in “Amigos de Fibro (Fibro Friends)”. The invited professionals will be as follows: a physiotherapist, a physician, a psychologist, a nutritionist, a nurse, a pharmacist, a speech therapist, an occupational therapist, a social worker, and a naturologist.

The activities will be conducted through an online group. In health promotion, group work makes it possible to break the vertical relationship that traditionally exists between the health professional and the subject of their action, which is a strategy that facilitates the expression of needs, expectations, anxieties, and life circumstances, which have some impact on the health of individuals and groups. Moreover, when developing the work with groups, the professional has the chance to stimulate participants to find collective strategies to confront the problems the community faces [32]. When starting a group, the uncertainties of the participants must be considered and, therefore, it is necessary to create a safe and reliable environment [33]. It is necessary to consider that, at this moment, introductions are formal and moments of silence are common [34]. It will be necessary to observe the group’s expectations and non-verbal communication, stimulate group cohesion and keep the group’s objectives clear, relating them to the members’ needs. At this time, it is also important to establish and maintain schedules, meeting locations and a limit of participants [34]. Participants will also be informed to avoid opening new tabs or web pages, other applications, and/or other distractions at the time of the meeting.

The language should be clear and easy to understand for the group at hand. These details can facilitate the group’s dynamics and good functioning and, consequently, obtain better results. Communication and empathy are also necessary [34]. Only effective communication can help the patient conceptualize their problems, face them, envision their participation in the experience, and alternative solutions for them, seeking to adapt to new patterns of behavior. Empathy refers to an emotional attunement of the coordinator with the participants—integrating into the group atmosphere [34].

Each meeting must be structuralized in, at least, three basic moments: an initial moment of group preparation for the work of the day (team-building exercises, warm-ups, relaxation); an intermediary, where the group is involved in a variety of activities that facilitate their reflection and elaboration of the developed subject; and a moment of systematization and evaluation of the work of the day that allows the participants to visualize their production as a workgroup [35]. In addition, this program will be based on the cognitive behavioral therapy model, which is one of the therapies that act on the effects regarding behavior, emotions, and symptoms. This approach has an emphasis on the current situation of the individual rather than on past situations [36–38]. Thoughts may represent cognitive changes that directly influence the health condition of the individual. In this sense, this therapy seeks to change the behavioral aspects of the individual by stimulating them to review inappropriate attitudes and beliefs that may negatively influence the health condition of these individuals [36–39].

Regarding the physical exercises that should be included in the treatment of individuals with fibromyalgia [10], a recent review on physical exercises for fibromyalgia published in the Cochrane Database of Systematic Review [40] highlights that no evidence was found of improvement in pain superior to that of the study by Assumpção et al. [41], where improvement was at 30%. In this sense, we chose to use the aforementioned protocol [41], which meets the guidelines of the European Alliance of Associations for Rheumatology [10], highlighting that for non-pharmacological interventions in the management of fibromyalgia, patient education is indicated, in association with physical exercise. Assumpção et al. [41] suggest the inclusion of both approaches in exercise therapy programs for fibromyalgia in clinical practice.

**Fig. 2:**
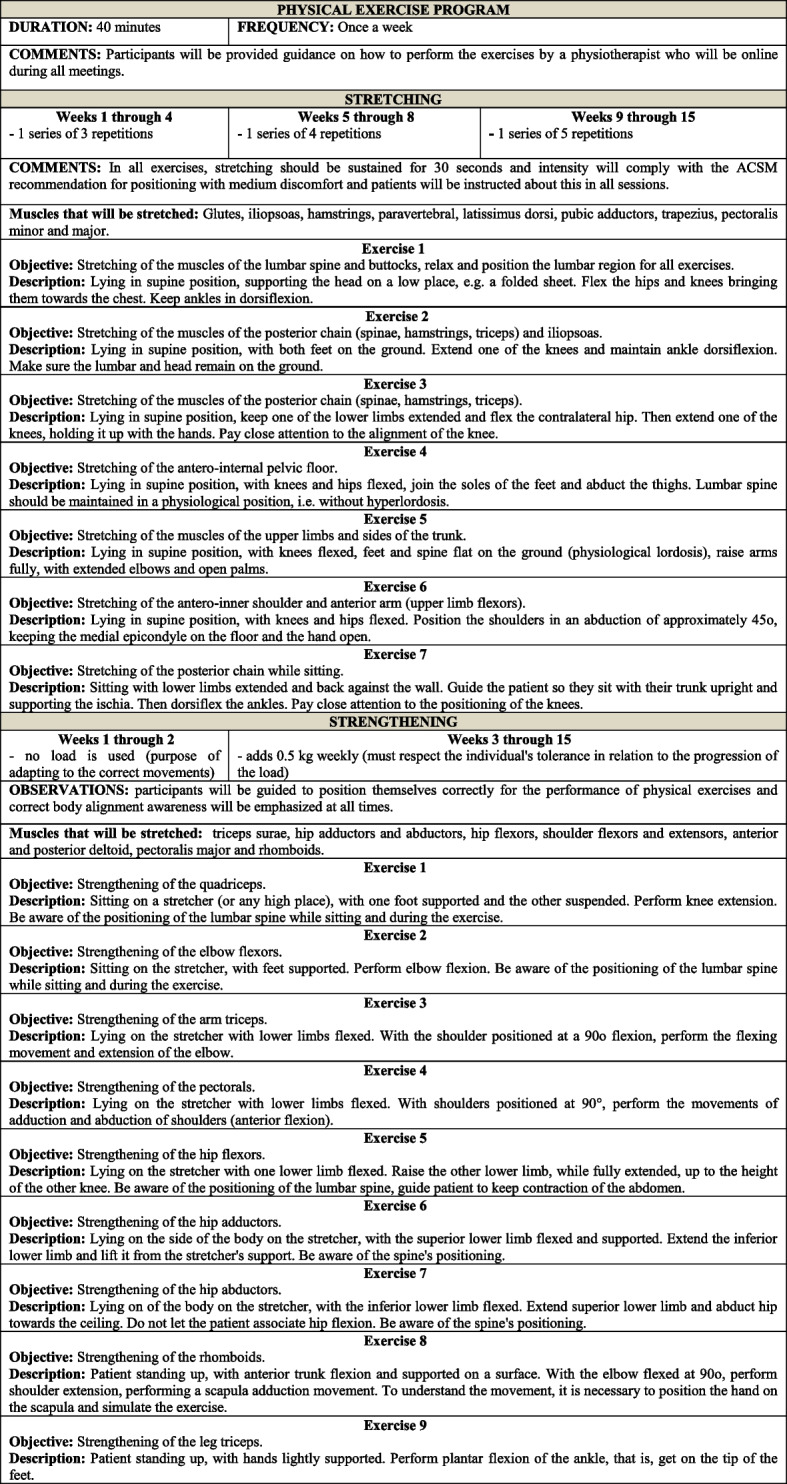
“Amigos de Fibro (Fibro Friends)” physical exercise program

Exercise sessions should be performed online with an approximate duration of 40 min each, once a week, for flexibility and low-intensity strengthening exercises [41]. Participants will be instructed to position themselves correctly to perform the exercises and, at all times, correct body alignment awareness will be emphasized [41]. The physical exercises will be interspersed: muscle stretching 1 week, and muscle strengthening the other. Participants will be tended to online as a group and under the guidance of a physiotherapist. Figure 2 presents the structure of the physical exercise program, based on Assumpção et al. [41] and the American College of Sports Medicine (ACSM) [42].

It is important to note that we considered the new version of guidelines on physical activity created by the World Health Organization in 2020 [43]. In this sense, the current guidelines recommend that all adults and elderly individuals with chronic conditions should perform at least 150 to 300 min of moderate-intensity aerial physical activity; or at least 75 to 150 min of vigorous-intensity aerial physical activity; or an equivalent combination of moderate-intensity and vigorous-intensity physical activities throughout the week for substantial health benefits [43]. So, considering that fibromyalgia is a chronic pain syndrome that has an important negative impact on the quality of life of patients, all participants will be instructed on the frequency and time of physical activities that should be performed per week and encouraged to perform them at home, in addition to what was proposed in the web-based program, to ensure the benefits of regular physical activity [43].

**Fig. 3:**
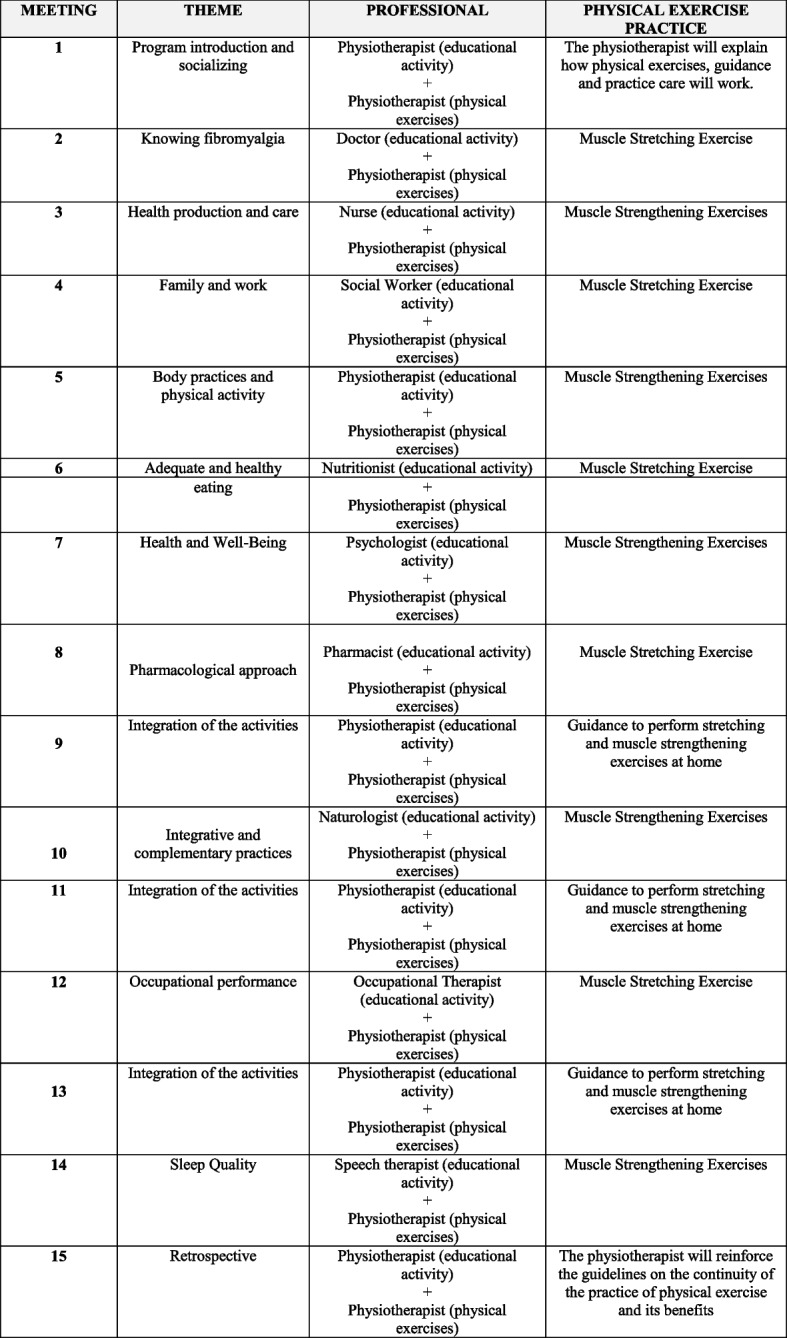
Script of “Amigos de Fibro (Fibro Friends)”

The “Amigos de Fibro (Fibro Friends)” program will be conducted by the invited professionals, according to the themes of each meeting that were developed in the first phase of the study, based on the National Health Promotion Policy [44], and is presented in Fig. 3. The development of “Amigos de Fibro (Fibro Friends)” was published and showed a good level of agreement among evaluators [45].

#### Control group

Participants in the CG will receive an educational self-care e-book that was prepared by the authors themselves and that will address the same information that was defined in the construction of “Amigos de Fibro (Fibro Friends)”. The order of the themes of the e-book will be the same as presented in the experimental group. The e-book will have weekly chapters, meaning each week you should read a chapter with the information and note the start and end date of the reading. The choice of weekly chapters is for the CG to finish participating in the study at the same time as the experimental group. Moreover, once a week, the responsible physiotherapist will call each participant of this group to ask how the reading is going and to remind them to carry it through weekly.

The final version of the e-book is comprised of 90 pages, with the title “Amigos de Fibro (Fibro Friends)”: Promoting Health in Fibromyalgia”. The e-book was created using the Canva software, a graphic design platform that allows users to create different types of visual content. In addition, it features graphic illustrations taken from Envato Elements, a subscription platform for graphic design items. The material was divided into 10 blocks with the following themes: getting to know fibromyalgia (chapter 1), health promotion and care (chapter 2), family and work (chapter 3), body practices and physical activities (chapter 4), healthy and adequate nutrition (chapter 5), health and well-being (chapter 6), pharmacological approach (chapter 7), integrative and complementary practices (chapter 8), occupational performance (chapter 9), and sleep quality (chapter 10).

The educational content refers to self-care measures that promote the health and quality of life of individuals with fibromyalgia. In order to enrich the content, improve readability and general reader captivation of the e-book, and offer the target audience the opportunity to delve deeper into the topic, references, and links were added to the end of the material, which give access to the entirety of articles, books, theses, and websites that also support the material’s theoretical framework. The e-book has already been validated and published and showed a good level of agreement among evaluators [31]. The final version of the e-book is available at http://www.amigosdefibro.com.br.

### Criteria for discontinuing or modifying allocated interventions {11b}

There will be no special criteria for discontinuing or modifying allocated interventions.

### Strategies to improve adherence to interventions {11c}

Once a week, the responsible researcher will call each participant to ask how the reading is going and to remind them to do it weekly (control group). In the intervention group, adherence will be analyzed and, if necessary, the researcher in charge will also call to find out the reason for the absences from the intervention.

### Relevant concomitant care permitted or prohibited during the trial {11d}

Implementation of the control group with a self-care educational e-book versus web-based educational intervention “Amigos de Fibro (Fibro Friends)” will not require change in usual care pathways (including the use of any medication) for the treatment of fibromyalgia and these will continue for both study arms.

### Provisions for post-trial care {30}

At the end of the project (end of the 12 months of follow-up), all participants will be invited to participate in the groups in which they were not selected, in order to thank all participants in the research.

### Outcomes {12}

Participants will be evaluated pre- and post-intervention by a blind evaluator, previously trained to obtain information and confirm eligibility criteria, in addition to primary and secondary outcomes. The evaluator will be asked to guess which group the participants were in (intervention or control) at the end of the study to measure the blindness of the evaluator. Due to the nature of the interventions, it will not be possible to blind neither the responsible participants nor professionals towards the application of the web-based intervention.

#### Main outcome variable

##### Quality of life

The Fibromyalgia Impact Questionnaire (FIQ) was first developed by Burckardt, Clark, and Bennett [46], and translated and adapted to the Brazilian population by Marques et al. [47], reviewed by Bennett et al. [48] and validated for Brazil by Paiva et al. [49] will be used. This tool has been demonstrated to be trustworthy and valid. The FIQ is a change-sensitive and clinically measurable tool [50], which has good discriminative power, with high sensitivity and specificities (78.2 and 81.2%, respectively) [51]. Other aspects of these tools are also important, such as applicability, practicality, and clarity [50]. This tool consists of 19 questions, organized into 10 items: functional capacity, well-being, work absences, workability, pain, fatigue, morning tiredness, stiffness, anxiety, and depression [47]. Item 1’s value can range from 0 to 30, and items 2 and 3’s values can range from 0 to 7. The remaining items are composed of visual analog scales ranging from 0 to 10. To obtain the tool’s score, item 2 must be recorded (0–7, 1–6, 2 = 5, 3 = 4, 4 = 3, 5 = 2, 6 = 1, and 7 = 0) and normalized on a scale of 0 to 10. Items 1 and 2 must also be normalized, so that all the items are expressed in similar units [47]. The total FIQ score will be obtained by the sum of the items, ranging from 0 to 100. The highest values indicate a greater impact of fibromyalgia in quality of life. The total score will also classify impairment as mild (0 to 38) or severe (59 to 100) [47].

#### Other variables

##### Socio-demographic and health profile

For the characterization of the sociodemographic and health profile, a semi-structured questionnaire will be used, composed of information related to age, sex, marital status, race, years of study, retirement, profession, monthly income in the minimum wage (R$ 1,212.00 for 2022) referring the 2010 Demographic Census of the Brazilian Institute of Geography and Statistics (IBGE) for smoking, self-perception of health status, and the number of medications used and morbidities.

##### Pain intensity

Pain intensity will be evaluated by the visual analog scale (VAS), which consists of a widely used instrument [52], with great discrimination power to evaluate individuals with fibromyalgia [53]. VAS is reliable and highly correlated with other forms of pain assessment [54]. It is sensitive to pharmacological and non-pharmacological procedures, which alter the painful experience, and strongly correlates with pain measured by other scales, since it has shown considerable discriminative power for pain detection in individuals with fibromyalgia, with high sensitivity and specificity (80 and 80%, respectively) [53]. This instrument consists of a straight 10-cm line, devoid of numbers, with indications at each end for “absence of pain” and “unbearable pain”. The individual marks on the line the intensity of their pain, which can range from 0 to 10 [55].

##### Sleep quality

For the evaluation of the quality of sleep, the Pittsburg Sleep Quality Index (PSQI) will be used. It is a tool of previously established trustworthiness and validity [56, 57]. Reliability estimates showed internal consistency of 0.83 for the seven component scores and test–retest reliability of 0.85 for the overall PSQI scores [58], presenting good applicability and effectiveness in clinical practice and research [59]. The discriminative power with sensitivity and specificities are considered high (89.6 and 86.5%, respectively) [60]. The instrument was created by Buysse et al. [60]. In its translated version, it was adapted and validated for the Brazilian population by Bertolazzi [61]. It is a self-report tool, which evaluates sleep quality in the last month, through 19 items, distributed between seven components (sleep quality, sleep latency, sleep duration, sleep efficiency, sleep alterations, use of medications for sleep and diurnal dysfunction), graded on a scale of 0 to 3. The sum of the components of the PSQI indicates the overall score, which ranges from 0 to 21. Scores between 0 and 4 suggest good sleep quality, between 5 and 10 indicate poor sleep quality, and above 10 points indicate the presence of a sleep disorder [62].

##### Self-care agency

Self-care agency will be evaluated by the Revised Self-Care Capacity Assessment Scale (R-SCAS), which was developed by Evers [63] and adapted and validated for the Brazilian context by Damásio and Koller [64]. This instrument presents good applicability [65], with an internal consistency classified as positive [66] and satisfactory results of validity and trustworthiness [65, 67]. The R-SCAS presents 15 items that correspond to a 5-point Likert scale, whose score ranges from 1 to 5, and contains statements that can be assigned five response options: (1) strongly disagree; (2) disagree; (3) neither disagree nor agree; (4) agree; (5) strongly agree. The total score of the R-SCAS is obtained by adding the results of the items and ranges from 15 to 75 points. Items 4, 11, 14, and 15 present negative content and their score needs to be reversed to obtain the total score. Higher values indicate greater self-care capacity [64].

##### Use and costs of health services and social assistance

The Client Socio-Demographic and Service Receipt Inventory (CSSRI) was developed in England by Chisholm et al. [68], to be used in economic studies and in studies that evaluate health services. This tool has been widely used in research in European countries and is a useful and reliable tool for this purpose. Since the structure of the health services offered varies in each country and region, local adaptation becomes mandatory. Thus, the CSSRI was translated into Portuguese and adapted to the organizational structure of public health services Brazil by Souza et al. [2]. The trustworthiness between appraisers was excellent as a whole [69–71]. This is a semi-structured inventory, divided into six main topics: (1) Sociodemographic data of users; (2) Living and housing conditions; (3) Use of community or hospital accommodations; (4) Employment and income data; (5) Medication consumption; (6) Use of health, social and criminal services. All information is referred to in a retrospective time period perspective (last month, last 3 months, last 6 months). This tool was adapted for the present study, using only data related to medication consumption and use of health and social services. The costs per unit for each of these services, the procedures performed and the medications consumed will be calculated later, based on the data collected [72].

##### Program feasibility

At the end of the feasibility study period, participants with fibromyalgia and professionals will make an evaluation with the researchers, of a quantitative and qualitative nature, with questions about the experience, themes, duration of the meeting, the performance of the professional, and the limitations encountered during the participation of the program. In addition, participants will also be instructed to record the level of satisfaction with the program using a 10-point Likert scale, where 0 represents totally dissatisfied and 10 represents totally satisfied [73]. Moreover, the expectation and the credibility of the result of the treatment will be evaluated by the Credibility/Expectancy Questionnaire (CEQ). The first and so far one of the most used tools for this variable [74, 75], so named by Devilly and Borkovec [76] and developed from a questionnaire based on, and created by, Borkovec and Nau [77], this tool evaluates the willingness of patients to recommend treatment to other people [75], as well as the expectations of the intervention [78]. The tool was adapted, translated into Portuguese, and used by previous Brazilian studies [79, 80]. Evidence of the good reliability and predictive validity of this measure was provided [76]. The CEQ is a scale divided into two types of questions [74]: four (items 1, 2, 3, and 5), which assess the expectation, that is, what they expect in relation to what the treatment improves (ranging 1 to 9) and two (items 4 and 6) assessing the credibility (ranging 0 to 100%). Both subscales have shown high internal consistency [76, 81]. Everything will be recorded and later analyzed by the physiotherapist responsible for identifying the problems and proposing solutions.

##### Program adhesion

At the end of the program, the participants’ adherence to “Amigos de Fibro (Fibro Friends)” will be verified, using the following parameters: abandonment rate (%) and attendance rate (%). These parameters also allow us to evaluate the viability of the program [73].

##### Qualitative process evaluation

For qualitative data, we will use a semi-structured interview script, composed of questions created by the authors themselves that deal with their participation, perception of the methodology, subjects addressed, and impact on their lives. The data collection process will be carried out individually by the researcher, who will schedule an appointment with each participant. There will also be the same questions for professionals. Data will be obtained through semi-structured interviews, recorded with the consent of the participants. The recordings will be transcribed by a company specialized in shorthand monitoring and audio transcription, providing reliability and faithfully revealing the testimonies of the participants.

Regarding the deadlines for pre and post evaluations, and interventions will be as follows:

- Pre-assessment: from 1 to 7 days before the intervention.
- Post-assessment: from 1 to 7 days after the intervention.
- Intervention: 3 months of intervention.
- Follow-up: 3, 6, and 12 months after the intervention.

### Participant timeline {13}

**Fig. 4:**
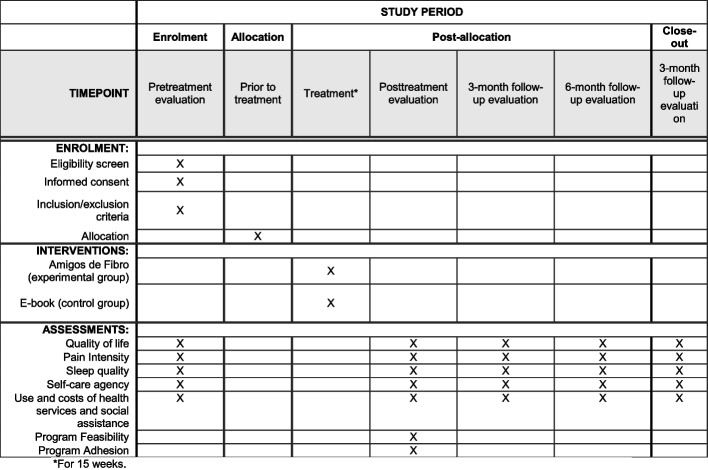
Study timepoints

The evaluation schedule, interventions, and follow-up are presented in Fig. 4 through a schematic diagram highly recommended in protocol studies.

### Sample size {14}

The sample size was based on the assumptions of group educational interventions, where it is not necessary to formally calculate the sample size due to the characteristics of the intervention [27, 38, 78, 82–84]. The size of the group should consider that the number of participants allows everyone to manifest and feel tended to. The coordinator should feel comfortable with the number of individuals and that the main needs of the participants are being met. Its size cannot exceed the limit that endangers visual and auditory communication [34, 85, 86]. It is recommended that the ideal size of a health education group be a maximum of 12 members [34]. The sample will consist of 24 participants, divided into two groups, with 12 individuals each. Regarding the loss of participants, we will use the intent-to-treat purpose. The present study hopes to obtain quantitative data to use as a basis for future studies of “Amigos de Fibro (Fibro Friends)”.

### Recruitment {15}

Recruitment is carried out through dissemination on the Brazilian Association Fibromyalgia (ABRAFIBRO) social networks, a support network for people seeking information about the syndrome in Brazil. The association has a supportive purpose and it is not possible to calculate the number of people who will receive the message, because social networks are comprehensive and allow the sharing of information. They will also be able to recruit people from rheumatology clinics throughout Brazil (all states); however, there is no exact number of rheumatology clinics in the country. Finally, as extra steps taken to recruit enough people, you may send letters to potential participants known to the research group. The estimated recruitment rate will be 80%.

## Assignment of interventions: allocation

### Sequence generation {16a}

Simple controlled randomization will be performed using Randomized (http://randomized.com), by an independent researcher, who will not be involved in the recruitment and intervention of participants.

### Concealment mechanism {16b}

The allocation will be made secretly, that is, through a numerical sequence, in which there will be a letter on whether the individual will belong to the experimental group (EG) or the control group (GC) [87].

### Implementation {16c}

An independent researcher who is not participating in the other stages of the study will be responsible for managing the allocation sequence, and enrollment of participants and will assign participants to interventions.

## Assignment of interventions: blinding

### Who will be blinded {17a}

Participants will be evaluated pre- and post-intervention by a blind evaluator, previously trained to obtain information and confirm eligibility criteria, in addition to primary and secondary outcomes. The evaluator will be asked to guess which group the participants were in (intervention or control) at the end of the study to measure the blindness of the evaluator. Due to the nature of the interventions, it will not be possible to blind neither the responsible participants nor professionals towards the application of the web-based intervention. Regarding the data analyst, he will be blind to carry out this stage of the study.

### Procedure for unblinding if needed {17b}

We believe that this procedure will not be necessary. If yes, we will take all necessary ethical measures. The circumstances in which the disclosure will be necessary may be as follows: there is a conversation between the participants in which intervention they are in, disclosure of photos or videos of the intervention posted by the participants on social networks, the evaluator for some personal reason revealing which group the participants are in, among other relevant ones.

## Data collection and management

### Plans for assessment and collection of outcomes {18a}

Data will be collected by only one trained person (pre, post, and follow-up) for standardization and to avoid data duplication. The person trained to collect the data is a researcher, with training in physiotherapy and a master’s degree in the area of health promotion. In addition, the person responsible for cleaving the data is not part of the authorship and group of researchers of the study. The instruments used in the study are detailed in the methodology section. All instruments are reliable and validated. If the reader needs a detailed version of the questionnaires, with all the tests, he can request the corresponding author by email and he will receive the file (in addition to the data that are explained in the protocol).

### Plans to promote participant retention and complete follow-up {18b}

After the intervention, the primary and secondary variables of both groups will be collected again, as well as after 3, 6, and 12 months to verify the effect and maintenance of the intervention. The researchers will be in contact with the patients on a monthly basis, sending positive messages on their phones to maintain a bond until the last evaluation.

### Data management {19}

Data will be collected through online forms, through the Google Forms platform. After data collection, participants will be identified by a number and names and private data will not be identified. Soon after, the data will be stored in Microsoft Office Excel (2013) with complete security. Data that are not in the protocol can be found through a direct request with the responsible author. There will be no paper-based data entry, as it will be electronic, as previously mentioned (Google Forms). Other important data management information will be:

- Simple controlled randomization will be performed using Microsoft Excel 8.0 software for Windows, by an independent researcher, who will not be involved in the recruitment and intervention of participants. The allocation will be made secretly, that is, through a numerical sequence in opaque and sealed envelopes, in which there will be a letter identifying which group the participant will be in.
- The participants will be evaluated individually pre and post-intervention by the same evaluator who did not know which group the participant belonged to. The evaluator will undergo a one-week training with a total of 15 h, based on the simulated application of the questionnaires, indicated verbal command and possible doubts that could arise during the application. After training, the evaluator will be able to obtain information and confirm the eligibility criteria, in addition to applying the evaluation instruments used.
- Data organization will also be carried out by an independent researcher.
- The data will be under the responsibility of the researchers and, if necessary, will be made available without identifying the participants.

### Confidentiality {27}

The study will adhere to the guidelines established by the General Law for the Protection of Personal Data, Law No. privacy. In accordance with the recommendation of the General Law for the Protection of Personal Data, data will be anonymized and password protected. In this context, data will be stored on a password-protected personal computer to prevent damage from occurring as a result of data processing. Participants will receive an individual study identification number. If requested, anonymous study data will be shared with other researchers to allow for prospective international meta-analyses. Also, according to item XI.2.f., of Resolution 466/12, the data will be kept in a digital file, under the custody and responsibility of the researcher, for a period of 5 years after the end of the research and will be available to any clarification of doubts.

### Plans for collection, laboratory evaluation, and storage of biological specimens for genetic or molecular analysis in this trial/future use {33}

There will be no collection of biological samples in the study.

## Statistical methods

### Statistical methods for primary and secondary outcomes {20a}

#### Quantitative analysis

Participant code numbers will ensure blinding of data analysis. Data will be entered into Microsoft Office Excel (2013) and will be used in the descriptive analysis of demographic data. Categorical data will be described using counts and percentages. As recommended, continuous data will be presented using medians and interquartile ranges, whether they be normal or not. Differences between intervention and control groups will be tested using non-parametric tests. A 5% level of significance will be used for all statistical tests. Data analysis will be undertaken in SPSS version 22.

#### Qualitative analysis

The data will be analyzed using the content analysis method proposed by Bardin [88], specifically the thematic content analysis, which allows the organization of textual content, to create categorizations and facilitate inferences and perceive patterns. Content analysis can be defined as a set of methodological instruments, in constant improvement, which lend themselves to analyzing different sources of content (verbal or non-verbal). This thematic analysis aims to discover the nuclei of meaning that constitute communication and whose presence may have some meaning for the chosen analytical objective, in addition to seeking answers to questions. With this technique, one can walk towards the discovery of what is hidden in the manifest contents, going beyond the appearances of what is being analyzed. Content analysis will be organized into three phases: (1) Pre-analysis (when you will listen to the audio and visualize the notes); (2) Exploration of the material (interview transcription); (3) Treatment of results: inference and interpretation (in this phase, the material will be read and re-read, in order to highlight relevant points and use a tool for visualization and tabulation of the most recurrent subjects in all the analyzed interviews). In this perspective, the next stage of the analysis will be the categorization of responses, according to a detailed and exhaustive study of the participants’ statements, which will enable the construction of categories to be discussed and analyzed.

The person responsible for analyzing the data will not know which group is control or intervention. In addition, data analysis will not be performed by a researcher from the study group.

### Interim analyses {21b}

If necessary, there will be intermediate reviews and termination guidelines. The responsible researcher will have access to this information and will make the decision.

### Methods for additional analyses (e.g., subgroup analyses) {20b}

There will be no further analysis. The analyses carried out are described in the study.

### Methods in analysis to handle protocol non-adherence and any statistical methods to handle missing data {20c}

In case of non-adherence of the participants, the intention-to-treat method will be used. The intent-to-treat method is a statistical approach used in clinical trials and experimental studies to analyze outcomes regardless of compliance with the original protocol. The principle of the intention-to-treat method is that participants should be analyzed according to the group to which they were initially assigned, regardless of whether or not they followed the treatment as planned.

### Plans to give access to the full protocol, participant-level data, and statistical code {31c}

The data will be made available to readers, if requested by email.

## Oversight and monitoring

### Composition of the coordinating center and trial steering committee {5d}

#### Coordination Center

- Study leaders will be available at any time to answer questions from participants.
- All will be available in the research lab and online communication networks.
- The group of advisors will be two (2) physiotherapist researchers responsible for the general direction of the study. One (1) rheumatologist responsible for the clinical aspects of the participants and one (1) person with fibromyalgia. All participants in this coordination group will be attentive to the event of the study. Weekly support may be requested. In addition, this group may meet to discuss the progress of the study every 15 days.

#### Trial steering committee

- A participant support committee with three members: one (1) physiotherapist, one (1) rheumatologist and one (1) patient with fibromyalgia. All three members of this committee have the role of managing and supervising all the steps of the study, as well as solutions for any problems that may exist.

#### Stakeholder and Public Involvement Group (SPIG)

- There will not be, as this study does not present commercial stakeholders, as well as the public. This is a research without conflict of interest.

### Composition of the data monitoring committee, its role and reporting structure {21a}

The Data Monitoring Committee will be composed of experts outside the study. Therefore, the following professionals will be part of it: 2 physiotherapists (analyzing data collection, ethical aspects with data management and data storage), 1 physician specialized in rheumatology (responsible for monitoring the applied instruments and how to use the data, as well as, care with the data of the participants) and a statistician (responsible for the storage and quality of the data). If it is necessary to write a report, these specialists will be responsible for this function and will send it to the researchers responsible for the study. In addition, all members of the Data Monitoring Committee are independent experts and have no connection with competing interests or sponsors/financiers.

### Adverse event reporting and harms {22}

#### Notification of adverse events (AEs)

The minor adverse events that can happen are falls during physical exercise. If any happens, it will be immediately communicated and verified by the Data Monitoring Committee.

#### Reporting of serious adverse events (SAEs)

Evidence suggests that SAEs are not expected, however, if any serious adverse effects occur, they will be immediately reported and analyzed by the Data Monitoring Committee.

#### Notification of intervention harm

In some situations, research may have unplanned or unforeseen harm or consequences, which may result in collateral damage or unwanted effects. If any of these happen, they will be immediately communicated and verified by the Data Monitoring Committee.

Participants will have an online communication channel (telephone and email) to fill out a form highlighting adverse effects and harm. A secretary will forward the message to the Data Monitoring Committee.

### Frequency and plans for auditing trial conduct {23}

#### Project management group

This is a specific team designated to plan, coordinate and supervise all activities related to the clinical trial. This group is essential to ensure that the trial is conducted properly, efficiently, and in compliance with established protocols and regulations for clinical research. The main responsibilities of the Project Management Group will include planning and organization, selection of centers and researchers, resource management, coordination and communication, monitoring progress, data management, risk management, and reporting and documentation. The joint work of the Project Management Group will be carried out by a group of 4 independent physiotherapists. Meetings will be online every 20 days.

#### Trial steering group

This group will represent a committee responsible for the supervision and general direction of the study. The main objective will be to provide an independent and unbiased review of the progress of the clinical trial, monitor the safety of participants, and review any issues or challenges that may arise during the development of the trial. The main responsibilities of this group will be as follows: general supervision of the study, regularly reviewing the progress of the study and its compliance with the research protocol; evaluation and approval of any relevant changes to the trial protocol, such as modifications to the inclusion/exclusion criteria or the method of data collection; ongoing monitoring of the ethical aspects of the study and ensuring that the study is being conducted in accordance with ethical principles and applicable regulations; participant safety assessment and review of any study-related adverse events; making important study-related decisions, such as continuation, modification, or early termination of the study; communicating with the research ethics committee and other regulatory authorities as needed; and finally, providing guidance and support to investigators and clinical trial staff. Membership of the Trial Steering Group will be carried out by a group of one (1) Physiotherapist, one (1) specialist in rheumatology, and one (1) patient with fibromyalgia. All three members will be independent. Meetings will be online every 20 days.

#### Independent data monitoring and ethics committee

This committee will play a crucial role in monitoring and overseeing the study. This committee will be independent because it operates separately from the research team conducting the study and the sponsors or interested parties involved. Its main objective is to protect the well-being and rights of study participants and to ensure the integrity and validity of research data. The main responsibilities of the Independent Data Monitoring and Ethics Committee include data monitoring, interim analysis, participant safety, ethical considerations, continuation or termination of the trial, and ultimately, the confidentiality and integrity of the data. This independent committee will be composed of 3 independent physiotherapists. Meetings will be online every 20 days.

### Plans for communicating important protocol amendments to relevant parties (e.g., trial participants, ethical committees) {25}

The communication of important changes to the protocol to the relevant parties will be carried out through a report and sent to the communication channels of the bodies involved.

### Dissemination plans {31a}

The dissemination plan will be through publications on institutional websites, television, radio, and podcast. We are going to hold an event to demonstrate the importance of the topic in clinical practice and encourage professionals to include “Amigos de Fibro (Fibro Friends)” in the clinical routine of primary health care throughout Brazil.

## Discussion

Due to the chronic nature of fibromyalgia, patients have a negative approach towards this syndrome and its consequences. To face this challenge, there has been a stimulus for the development of educational programs with the aim of promoting the health of affected individuals. These programs aim to improve care through guidance provided by professionals from different areas of knowledge, who instruct patients on how to manage pain and deal with problems related to their lifestyle. These professionals collaborate with each other to create a practical care approach, considering multi- and interdisciplinary perspectives, bringing together diverse knowledge and perspectives [18].

Our study aims to evaluate the impact of a web-based educational intervention on the quality of life of patients with fibromyalgia. The results of our study may, therefore, contribute with currently insufficient data on the non-pharmacological clinical management of fibromyalgia in Brazil. Previous interventions that used health education only as control groups in people with fibromyalgia suggest evidence that health education techniques should be used in association with other interventions [16–18].

To improve understanding the impact of the intervention on the life of patients with fibromyalgia, we will use different methods of data collection and analysis. For this reason, we will conduct semi-structured qualitative interviews with participants from both groups, so that we can interpret our quantitative findings in the context of participants’ experiences at the individual level.

The meetings will be organized by the assigned physiotherapist and a guest from the thematic field of the meeting. The invited professionals will be experts in rheumatology and health promotion. The involvement of the public and patients in the research is associated with better results and translation into practice, and is advocated by the European Alliance of Associations for Rheumatology [89].

“Amigos de Fibro (Fibro Friends)” began to be shown in national and international scientific literature, as well as in scientific events with the aim of disseminating scientific knowledge and the application of “Amigos de Fibro (Fibro Friends)” in different spaces and contexts. In addition, the great project “Amigos de Fibro (Fibro Friends)” has already published its development and validation version in national and international journals, as well as presented at various events related to rheumatology and public health [90–95].

Our study presents as a limitation the non-blinding of the professional who will apply the intervention, as well as of the participant, because it is not possible to blind them to the type of intervention. We hope that the results of this study will help standardize health education interventions and provide positive evidence of the effectiveness of “Amigos de Fibro (Fibro Friends)” in patients with fibromyalgia.

### Practical implications

The study will generate qualified information for the health system that will serve as support to guide the effectiveness, evaluation, and sustainability of an educational program to promote health for individuals with fibromyalgia in Brazil. The aim is to present to primary health care managers the importance of including this program in the routine of Brazil’s Basic Health Unit to strengthen health promotion actions. Moreover, in recent years, organizations have been concerned and involved in the debate regarding the Objectives of Sustainable Development. In this sense, they are encouraged to create alternatives that allow the development of different ways to promote the quality of life of the population through effective and sustainable actions. The relevance of this work is linked to the 3rd objective, which seeks to guarantee access to quality health and promote well-being for all, at all ages.

## Conclusions

Future studies aimed at promoting the health of patients with fibromyalgia through web-based educational approaches may follow this methodology. The aim of this study will be to describe the appropriate procedure to obtaining a valid, consistent and reliable tool to be used during treatment in patients with fibromyalgia. The development and viability of “Amigos de Fibro (Fibro Friends)” will provide the scientific community with a tool that covers different aspects that are interfered with in fibromyalgia, and the self-care measures available to promote the health of fibromyalgia patients. Thus, this study will allow considering the use of this light technology tool in health in the future by several professionals working in Primary Health Care.

## Trial status

Protocol version number and date: version 1.

Recruitment start date: September 1, 2023.

Approximate date on which recruitment will be completed: October 30, 2023.

## Data Availability

The data will be made available to readers, if requested by email.

## Abbreviations

ABRAFIBRO: Brazilian Fibromyalgia Association
ACSM: American College of Sports Medicine
CEQ: Credibility/Expectancy Questionnaire
CONSORT: Consolidated Standards of Reporting Trials Statement
COREQ: Consolidated Criteria for Reporting Qualitative Research
CSSRI: The Client Socio-Demographic and Service Receipt Inventory
EG: Experimental Group
EULAR: European Alliance of Associations for Rheumatology
FIQ: The Fibromyalgia Impact Questionnaire
GC: Control Group
IBGE: Brazilian Institute of Geography and Statistics
ICD-10: Statistical Classification of Diseases and Related Health Problems
PSQI: Pittsburg Sleep Quality Index
R-SCAS: Revised Self-Care Capacity Assessment Scale
SCIELO: Scientific Electronic Library Online
VAS: Visual analog scale
